# The time is now: Achieving FH paediatric screening across Europe – The Prague Declaration

**DOI:** 10.3205/hta000136

**Published:** 2022-09-30

**Authors:** Nicole Bedlington, Marianne Abifadel, Birgit Beger, Mafalda Bourbon, Héctor Bueno, Richard Ceska, Kristýna Cillíková, Zdenka Cimická, Magdalena Daccord, Carine de Beaufort, Kanika I. Dharmayat, Brian A. Ference, Tomáš Freiberger, Marius Geanta, Samuel S. Gidding, Urh Grošelj, Martin Halle, Neil Johnson, Tanja Novakovic, Ondrej Májek, Athanasios Pallidis, Noel Peretti, Fausto J. Pinto, Kausik Kosh Ray, Bleddyn Rees, John Reeve, Željko Reiner, Raul D. Santos, Heribert Schunkert, Jaka Šikonja, Milka Sokolovic, Lale Tokgözoglu, Michal Vrablík, Albert Wiegman, Iñaki Gutiérrez-Ibarluzea

**Affiliations:** 1 FH Europe, Star House, Rochester, UK; 2 Laboratory of Biochemistry & Molecular Therapeutics, Faculty of Pharmacy, Pôle Technologie–Santé, Saint Joseph University of Beirut, Beirut, Lebanon; 3 Laboratory for Vascular Translational Science, Paris Cité University, Paris, France; 4 Sorbonne Paris Nord University, INSERM, Paris, France; 5 European Heart Network, European Alliance for Cardiovascular Health, Brussels, Belgium; 6 Unidade de I&D, Grupo de Investigação Cardiovascular, Departamento de Promoção da Saúde e Prevenção de Doenças Não Transmissíveis, Instituto Nacional de Saúde Doutor Ricardo Jorge, Lisboa, Portugal; 7 BioISI – Biosystems & Integrative Sciences Institute, Faculdade de Ciências, Universidade de Lisboa, Lisboa, Portugal; 8 Centro Nacional de Investigaciones Cardiovasculares, Madrid, Spain; 9 Cardiology Department, Hospital Universitario 12 de Octubre, Madrid, Spain; 10 Instituto de Investigación Sanitaria Hospital 12 de Octubre (imas12), Madrid, Spain; 11 Centro de Investigación Biomédica en Red Enfermedades Cardiovaculares, Madrid, Spain; 12 Facultad de Medicina, Universidad Complutense de Madrid, Madrid, Spain; 13 3rd Department of Medicine – Department of Endocrinology & Metabolism of the 1st Faculty of Medicine, Charles University & General University Hospital, Prague, Czech Rep; 14 Diagnoza FH, Libáň, Czech Rep; 15 Diabetes & Endocrine Care Clinique Pediatrique/CHL, Luxembourg, Grand Duchy Luxembourg; 16 Imperial Centre for Cardiovascular Disease Prevention, Department of Primary Care and Public Health, Imperial College London, London, UK; 17 University Of Cambridge, Clinical School, Cambridge, UK; 18 Centre for Cardiovascular Surgery and Transplantation, Brno, Czech Rep; 19 Medical Faculty, Masaryk University, Brno, Czech Rep; 20 Department of Endocrinology, Diabetes & Metabolic Diseases, University Children’s Hospital, University Medical Centre Ljubljana, Ljubljana, Slovenia; 21 Faculty of Medicine, University of Ljubljana, Ljubljana, Slovenia; 22 Department of Prevention & Sports Medicine, University Hospital Klinikum rechts der Isar, Technical University of Munich, Munich, Germany; 23 Deutsches Zentrum für Herz-Kreislauf-Forschung, Munich Heart Alliance, Munich, Germany; 24 Global Heart Hub, Galway, Ireland; 25 ZEM Solutions Ltd, Belgrade, Serbia; 26 National Screening Centre, Institute of Health Information & Statistics of the Czech Republic, Prague, Czech Rep; 27 Institute of Biostatistics & Analyses, Faculty of Medicine, Masaryk University, Brno, Czech Rep; 28 LDL Greece, Athens, Greece; 29 Department of Pediatric Gastroenterology-Hepatology & Nutrition, Hospices Civil de Lyon HCL, Hôpital Femme Mere Enfant HFME, Bron, France; 30 Univ-Lyon, CarMeN laboratory, INSERM U1060, INRAE U1397, Université Claude Bernard Lyon-1, Oullins, Lyon, France; 31 World Heart Federation, Geneva, Switzerland; 32 Cardiovascular Department, CCUL, CAML, Lisbon School of Medicine, University of Lisbon, Lisbon, Portugal; 33 European Atherosclerosis Society, Göteborg, Sweden; 34 The Digital Health Society, Dublin, Ireland; 35 The European Connected Health Alliance, Belfast, UK; 36 University Hospital Center Zagreb, Department for Metabolic Diseases, Zagreb, Croatia; 37 Lipid Clinic Heart Institute, University of Sao Paulo Medical School Hospital, Sao Paulo, Brazil; 38 Hospital Israelita Albert Einstein, Sao Paulo, Brazil; 39 International Atherosclerosis Society, Milan, Italy; 40 Deutsches Herzzentrum München, Munich, Germany; 41 Europan Public Health Alliance, Brussels, Belgium; 42 Department of Cardiology, Hacettepe University, Ankara, Turkey; 43 3rd Department of Internal Medicine, General Teaching Hospital, Prague, Czech Rep; 44 1st Faculty of Medicine, Charles University, Prague, Czech Rep; 45 University of Amsterdam, Department of Paediatrics, Amsterdam, Netherlands; 46 Basque Foundation for Health Innovation and Research, Barakaldo, Spain

**Keywords:** familial hypercholesterolaemia, FH, screening, children, cardiovascular disease, CVD, prevention, cardiovascular health, CVH, declaration, public health, health systems, EU, Europe, EU best practice, implementation

## Abstract

Familial hypercholesterolaemia (FH) is the most common inherited metabolic disorder characterized by high cholesterol and if left untreated leads to premature cardiovascular disease, such as heart attacks. Treatment that begins early in life, particularly in childhood, is highly efficacious in preventing cardiovascular disease and cost-effective, thus early detection of FH is crucial. However, in Europe, less than 10% of people living with FH are diagnosed and even less receive life-saving treatment. The Prague Declaration is a call to action for national and European Union policymakers and decision-makers and a result of the Czech EU Presidency meeting on FH Paediatric Screening (early detection of inherited high cholesterol) at the Czech Senate in Prague on 6th September 2022. It builds on a considerable body of evidence which was discussed at the Technical Meeting under the auspices of the Slovenian EU Presidency in October 2021. The Prague meeting addressed the outstanding barriers to the systematic implementation of FH paediatric screening across Europe. In this article, we present the key points from the Prague meeting and concrete actions needed to move forward.

## Preamble

Familial Hypercholesterolaemia (FH) is severely under-recognized, under-diagnosed and under-treated in Europe, leading to a significantly higher risk of premature cardiovascular diseases in those affected. FH stands for inherited, very high cholesterol and affects 1:300 individuals regardless of their age, race, sex, and lifestyle, making it the most common inherited metabolic disorder and a non-modifiable cardiovascular disease risk factor in the world.

With a 50% chance of inheriting the condition, every individual with an FH-causing variant also has at least one parent, often siblings, and sometimes children and grandchildren with the same vairant presenting a cardiovascular health burden for affected families. In Europe, there are over 500,000 children and 2,000,000 adults affected by FH. However, 5% of these children are identified and only a small fraction of all affected individuals receives life-saving treatment. Homozygous FH (HoFH) is the rare and the most severe form of FH. Untreated, HoFH often causes heart disease (heart attacks and aortic valve disease) in early childhood. As treatment beginning early in life is highly efficacious in preventing cardiovascular diseases in these individuals and cost-effective [1], [2], [3], early detection of FH is crucial.

The Prague Declaration is a call to action for national and European/international policymakers and decision-makers, building on a considerable body of quality evidence to date. It also reflects the outcomes of a meeting dedicated to FH paediatric screening under the auspices of the Czech EU Presidency on 6 September 2022, during which outstanding barriers to the implementation of FH paediatric screening systematically across Europe were addressed, bringing tangible solutions to move forward:

- Recognising the WHO Consultations on FH in 1998 which, for the first time, called on governments and public health systems to address the risk factors linked to inherited lipid disorders [4];
- Acknowledging that the Council of the European Union in December 2015 presented FH as “a medical model using characterization of individuals’ phenotypes and genotypes (e.g. molecular profiling, medical imaging, lifestyle data [5]);
- Recalling the Global Call for Action on FH published in 2020 under the title “Reducing the Clinical and Public Health Burden of Familial Hypercholesterolemia”, a global FH advocacy community effort of adopting the 9 (out of the initial 11) updated WHO public policy recommendations, covering awareness; advocacy; screening, testing, and diagnosis; treatment; family-based care; registries; research; and cost and value [6];
- Acknowledging that FH paediatric screening was recognized in 2021 by the European Commission Public Health Best Practice Portal as one of the best practices in non-communicable disease prevention [7];
- Noting the Lancet article published in September 2021 entitled “Familial Hypercholesterolaemia – too many lost opportunities” [8];
- Having regard to the high-level technical meeting under the Slovenian EU Presidency in October 2021 and the resulting scientific and political consensus on FH Paediatric Screening and recommendations therefrom [9], [10];
- Having regard to the World Heart Federation Whitepaper “Improving Prevention and Control of Raised Cholesterol” published in 2022, which references the importance of FH paediatric screening [11];
- Recognizing the lack of awareness among decision makers and citizens in general regarding the burden of FH, the need for knowledge building and a policy framework to make this disease a priority;
- Recognizing the European Commission’s “Healthier Together” Initiative addressing non-communicable diseases, including cardiovascular diseases [12];
- Welcoming the Czech EU Presidency’s focus on prevention and rare diseases, highly relevant in the context of HoFH, a rare form of FH [13].

## The FH European Community calls for

### 1. Political leadership and commitment to make FH paediatric screening a reality

Political leaders should commit to deliberate and bold efforts to make FH paediatric screening a reality in their country, acknowledging the unequivocal scientific evidence base, cost effectiveness analyses and a (children’s) rights’ perspective, in the spirit of leaving no-one behind.

A multi-stakeholder approach is also needed, to ensure health systems can integrate FH paediatric screening effectively. This includes enablers such as digitalisation, responsible health data sharing, and healthcare professionals (HCPs) education.

### 2. Investment and a policy framework for raising awareness of FH amongst medical practitioners and the public, to build trust and responsiveness

National governments in the EU should mobilise significant investment and create appropriate policy guidance to raise awareness amongst the public and healthcare professionals about FH and associated risks, applying the latest knowledge about personalised prevention, behavioural science, health literacy and the social determinants of health.

Awareness-raising campaigns and initiatives should be co-created through meaningful engagement of patient organisations, healthcare professionals and citizens.

### 3. Comprehensive early detection, screening, diagnosis and life course care programmes in every country

Every country should establish systematic early detection screening and diagnosis for FH, with an appropriate care programme focused on childhood identification and treatment.

The programme should be aligned with the “Best Practices on How to Establish a Screening Programme” [7] and in particular, to those defined by the European Commission’s public health best practice portal for FH.

Country/region-wide lipid referral centres should coordinate screening and promote family-based care.

Lipid referral centres should be guided by the experience of European Reference Networks and the European Atherosclerosis Society Lipid Clinics Network [14].

Each screening programme should incorporate universal, cascade or reverse cascade opportunistic strategies.

These may be based initially on cholesterol testing; however, FH genetic testing or ideally a combination of both should be developed as soon as feasible. 

They should be country/region-specific and in accordance with the organisational structure of respective health care systems. 

The screening could occur in the context of regular health care visits (such as vaccinations) or routine healthy children’s follow-up, in community settings, or around the perinatal period.

### 4. Specific actions to address the barriers to successful large-scale uptake of screening programmes and subsequent treatment

Positive action is needed to ensure the success of screening, including public information campaigns and personalised health advice. It is also important to ensure that a positive diagnosis has no adverse effects on access to treatment for patients and their immediate families.

Cost-benefit models should be developed that can be tailored to specific national scenarios to show the long-term cost advantages of FH screening as well as the benefits to individual citizens and their families of early diagnosis.

### 5. Targeted R&D to address knowledge gaps

Research to support childhood FH identification should include:

- new methods for early identification, diagnosis (including genetic diagnosis), personalised treatments and follow-up;
- registries that document FH care, monitor progress in achieving guideline-based treatment goals, and measure health outcomes. These should be developed in the context of the European Health Data Space and the European Reference network and in conjunction with the international FH registry [15];
- long-term clinical trials (3–5 years) and longitudinal studies in children and young people to further assess health outcomes, complications, cost-effectiveness, affordability, and feasibility, that allows full Health Technology Assessments to support decision-making processes;
- implementation of science to facilitate guideline-based FH care and assess citizen satisfaction with programmes;
- innovation in personalised prevention and treatment in FH;

New R&D should be supported through adaption of the appropriate policies.

### 6. Building the capacity of health professionals and empowering patients on how to best support individuals and families with FH

Based on the best available evidence and good practice, capacity-building programmes, and training materials should be created for professionals and individuals, to better deal with the pathology and its burden.

Dialogues should take place with patients and healthcare professionals to discuss last advances and evidence.

### 7. Commitment to shared learning and monitoring through exchange and comparisons beyond borders in- and outside the EU

Through funding programmes such as EU4Health, there should be investment in the transferability and uptake of best practice models in FH paediatric screening from other countries, and country level ‘score cards’ to measure progress according to safety, efficacy, cost and cost-effectiveness, organisational, ethical, legal and social criteria.

The experience of FH paediatric screening should be carefully observed and documented for analysis in the context of wider efforts towards better cardiovascular health (CVH) through collaboration with relevant national, European and Global alliances.

## Conclusions

We invite national and regional policymakers across the EU, medical societies, patient and public health organisations, and individual experts to support this declaration and to help ensure that FH Paediatric Screening becomes a reality in Europe, as part of European and national strategies to prevent cardiovascular diseases, and to promote cardiovascular health. 

## Note

The Time is Now: Achieving FH paediatric screening across Europe – The Prague Declaration was endorsed by the following organizations/institutions (see Attachment 1).

## Acknowledgements

FH Europe and Diagnoza FH would like to acknowledge and thank the Czech Deputy Minister for Health for endorsing the Prague Declaration and support for the FH Paediatric Screening meeting on 6 September, held under the auspices of Czech EU Presidency. We would also like to acknowledge and thank Senator Roman Kraus, Chair of the Senate Health Committee, for hosting the meeting at the Czech Senate and endorsing the Prague Declaration. 

We are especially indebted to all those who contributed to the research, writing, development, editing and design of this declaration. We would like to further acknowledge and thank all organisations, and individuals who have endorsed the Prague Declaration and their on-going support of FH Paediatric Screening efforts across Europe. 

## Conflict of interest

NB is a senior advisor to FH Europe. 

HB receives research funding from the Instituto de Salud Carlos III, Spain (PIE16/00021 & PI17/01799, PI21/01572), Sociedad Española de Cardiología, AstraZeneca, PhaseBio and Novartis; has received consulting/speaking fees from Astra-Zeneca, Novartis, Novo Nordisk and Organon: and is a scientific advisor for MEDSCAPE-the heart.og. 

KID reports grants from Pfizer, Amgen, Merck Sharp & Dohme, Sanofi–Aventis, Daiichi Sankyo, and Regeneron, during the conduct of the study; and personal fees from Bayer, outside the submitted work.

BAF received grants from Novartis, Amgen, Merck, Esperion Therapeutics, Pfizer; and personal fees for lectures, advisory boards, and/or consulting from Novartis, Amgen, Regeneron, Sanofi, Merck, AstraZeneca, Pfizer, Eli Lilly, Novo Nordisk, The Medicines Co, Mylan, Daiichi Sankyo, Viatris, Ionis Pharmaceuticals, dalCOR, CiVi Pharma, KrKa Phamaceuticals, American College of Cardiology, European Atherosclerosis Society, European Society of Cardiology.

TF is supported by the project National Institute for Research of Metabolic and Cardiovascular Diseases (Programme EXCELES, ID Project No. LX22NPO5104) – Funded by the European Union – Next Generation EU. TF received honoraria from Sanofi, Amgen and Novartis in the last 3 years, outside the submitted work.

SSG is a consultant at Esperion Therapeutics.

UG and part of preparatory work for the Declaration were supported by the Slovenian Research Agency (grants No. J3-2536 and P3-0343).

FJP reports grants and modest personal fees from Abbott, AstraZeneca, Boehringer Ingelheim, Bayer, Daiichi Sankyo, Novartis, Pfizer, Philips, Sanofi/Regeneron, Servier, Vifor, outside the submitted work.

KKR has received honoraria for consulting, lectures from Kowa, Amgen, Regeneron Pharmaceuticals, Sanofi, Daiichi Sankyo, Pfizer, Viatris, AstraZeneca, Eli Lilly, Esperion, New Amsterdam Pharma, Novartis, Silence Therapeutics, Bayer, Boehringer Ingelheim, Novo Nordisk, SCRIBE, CRISPR, Cargene, Vaxxinity, Abbott, Resverlogix. In addition, he has received research grant support to his institution from Sanofi, Daiichi Sankyo, Amgen, Pfizer and MSD

ŽR has received honoraria from Novartis.

RDS honoraria related to consulting, research and/or speaker activities from: Abbott, Ache, Amgen, Amryt, Astra Zeneca, Biolab, Esperion, Eli-Lilly, Getz Pharma, Hypera Farma, Kowa, Libbs, Novo-Nordisk, Novartis, Merck, Pfizer, PTC Therapeutics and Sanofi.

HS received honoraria and grants from MSD SHARP & DOHME, AMGEN, Bayer Vital GmbH, Boehringer Ingelheim, Daiichi Sankyo, Novartis, Servier, Brahms, Bristol-Myers Squibb, Medtronic, Sanofi Aventis, Synlab, Astra-Zeneca, Pfizer and Vifor.

LT received honoraria from the following companies: Abbott, Amgen, Bayer, Daiichi Sankyo, Janssen, Novartis, Novo Nordisk, Sanofi, Pfizer, Recordati.

AW reports research support for pharmaceutical trials of lipid-modifying agents from Amgen, Regeneron, Novartis and Silence Therapeutics.

Authors declaring no conflicts of interest: MA, BB, MB, RC, ZC, KC, MD, CdB, MG, MH, NJ, TN, OM, AP, NP, BR, JR, JŠ, MS, MV, IGI.

## Supplementary Material

Supplementary material – Endorsements
