# Supplementary material for: The time is now: Achieving FH paediatric screening across Europe – The Prague Declaration
Source: GMS Health Innov Technol. 2022 Sep 30;16:Doc04. doi: 10.3205/hta000136 (PMC9583732; doi:10.3205/hta000136)
Supplement: Supplementary material – Endorsements [file HInT-16-04-s-001.pdf]

Supplementary material – Endorsements

The Time is Now: Achieving FH paediatric screening across Europe – The Prague Declaration was endorsed by the following organizations/institutions:

International

- ECHAlliance - The European Connected Health Alliance Group, https://echalliance.com
- EPHA - European Public Health Alliance, https://epha.org
- EACH - European Alliance for Cardiovascular Health, https://www.cardiovascular-alliance.eu
- EAS FHSC - European Atherosclerosis Society FH Studies Collaboration, www.eas-society.org/page/fhsc
- EAS - European Atherosclerosis Society, www.eas-society.org
- EHN - European Heart Network, https://www.ehnheart.org
- FH Europe, The European FH Patient Network, www.fheurope.org
- Global Heart Hub, https://globalhearthub.org
- IAS - International Atherosclerosis Society, www.athero.org
- International HealthTechScan, https://www.i-hts.org
- ILEP - International Lipid Expert Panel, https://ilep.eu
- ISPAD - International Society for Pediatric and Adolescent Diabetes, https://www.ispad.org
- MEDizzy, https://medizzy.com
- ScreenPro FH, http://screenprofh.com
- The Digital Health Society, https://thedigitalhealthsociety.com
- WHF - World Heart Federation, www.world-heart-federation.org

Austria

- FHChol, https://fhchol.at

Bulgaria

- Bulgarian Hypertension League, http://www.hypertensionleaguebg.info/about-bhl

Cyprus

- Cyprus Atherosclerosis Society/CyFH Patient Registry, https://cas.org.cy/cyfh-info

Czech Republic

- Center of Cardiovascular Surgery and Transplantation Brno, https://www.cktch.cz
- Czech Society for Atherosclerosis, https://athero.cz
- Czech Society of Cardiology, http://www.kardio-cz.cz
- DIagnoza FH, https://diagnozafh.cz
- General University Hospital in Prague, https://www.vfn.cz
- Institute of Health Information and Statistics of the Czech Republic, https://www.uzis.cz
- Masaryk University, www.muni.cz

France

- NSFA - French Society of Atherosclerosis, https://www.nsfa.asso.fr
- ANHET.F - Association Nationale des Hypercholestérolémies familiales et Lipoprotéines(a), https://www.anhet.fr

Germany

- CholCo - Cholesterin & Co: Patientenorganisation für Patienten mit Familiärer Hypercholesterinämie oder anderen schweren genetischen Fettstoffwechselstörungen, www.cholco.de
- DigiMed Bayern, https://www.digimed-bayern.de

Germany, Austria, Switzerland

- D•A•CH-Gesellschaft Prävention von Herz-Kreislauf-Erkrankungen e.V., www.dach-praevention.eu

Greece

- LDL Greece, https://www.ldlgreece.gr

Hungary

- SZÍVSN / Heartily Hungary, https://szivsn.hu

Iraq

- ILCN - Iraqi Lipid Clinics Network, http://www.iraqilcn.com

Ireland

- Croí, The Heart and Stroke Charity, https://croi.ie

Italy

- LIPIGEN - Fondazione SISA, www.sisa.it/LIPIGEN

Latvia

- ParSirdi.lv, www.parsirdi.lv

Lithuania

- LŠA - Lithuanian Heart Association, www.heart.lt
- SVEIKA SIRDIS - The Healthy Heart, www.sveikasirdis.com

Norway

- NKT for FH, http://nktforfh.no

Poland

- Association of Patients with Family Hyperlipidemia in Gdańsk, https://www.facebook.com/hipercholesterolemia
- ECO Serce, Poland, http://ecoserce.pl
- KCHR - National Center for Family Hypercholesterolaemia, http://www.hipercholesterolemia.com.pl
- PTL - Polish Lipids Society, https://ptlipid.pl

Portugal

- National Institute of Health Doutor Ricardo Jorge, www.insa.min-saude.pt
- Portuguese Atherosclerosis Society, http://spaterosclerose.org

Romania

- InoMed - Centre for Innovation in Medicine, www.ino-med.ro

Slovakia

- MEDPED - Coordination Center for Familial Hyperlipidemias, Slovak Medical University, www.medpedfh.sk
- SAA - Slovak Association of Atherosclerosis, www.saa.sk
- Srdce rodiny,n.f, www.medpedfh.sk

Slovenia

- University Medical Centre Ljubljana, www.kclj.si
- ZA SRCE - Slovenian Heart Foundation, www.zasrce.si

Spain

- FHF - Fundación Hipercolesterolemia Familiar Spain, www.colesterolfamiliar.org

The Netherlands

- Amsterdam UMC, www.amc.nl
- LEEFH - Landreljik Expertisecentrum Erfeljikheidsonderzoek Familiare Hart, https://leefh.nl
- Stichting VrouwenHart, https://vrouwenhart.nl

Turkey

- Ailevi Hiperkolesterolemi dernegi

United Kingdom

- HEART UK, www.heartuk.org.uk

Attachement to*: Bedlington N, Abifadel M, Beger B, Bourbon M, Bueno H, Ceska R, Cillíková K, Cimická Z, Daccord M, de Beaufort C, Dharmayat KI, Ference BA, Freiberger T, Geanta M, Gidding SS, Grošelj U, Halle M, Johnson N, Novakovic T, Májek O, Pallidis A, Peretti N, Pinto FJ, Ray KK, Rees B, Reeve J, Reiner Ž, Santos RD, Schunkert H, Šikonja J, Sokolovic M, Tokgözoglu L, Vrablík M, Wiegman A, Gutiérrez-Ibarluzea I. The time is now: Achieving FH paediatric screening across Europe – The Prague Declaration. GMS Health Innov Technol. 2022;16:Doc04. DOI: 10.3205/hta000136, URN: urn:nbn:de:0183-hta0001369*

Attachement to: *Bedlington N, Abifadel M, Beger B, Bourbon M, Bueno H, Ceska R, Cillíková K, Cimická Z, Daccord M, de Beaufort C, Dharmayat KI, Ference BA, Freiberger T, Geanta M, Gidding SS, Grošelj U, Halle M, Johnson N, Novakovic T, Májek O, Pallidis A, Peretti N, Pinto FJ, Ray KK, Rees B, Reeve J, Reiner Ž, Santos RD, Schunkert H, Šikonja J, Sokolovic M, Tokgözoglu L, Vrablík M, Wiegman A, Gutiérrez-Ibarluzea I. The time is now: Achieving FH paediatric screening across Europe – The Prague Declaration. GMS Health Innov Technol. 2022;16:Doc04. DOI: 10.3205/hta000136, URN: urn:nbn:de:0183-hta0001369*
